# The effect of sodium nitrite infusion on renal function, brachial and central blood pressure during enzyme inhibition by allopurinol, enalapril or acetazolamide in healthy subjects: a randomized, double-blinded, placebo-controlled, crossover study

**DOI:** 10.1186/s12882-018-1035-x

**Published:** 2018-09-21

**Authors:** Jeppe B. Rosenbaek, Erling B. Pedersen, Jesper N. Bech

**Affiliations:** University Clinic in Nephrology and Hypertension, Regional Hospital West Jutland and Aarhus University, Laegaardvej 12J, DK-7500 Holstebro, Denmark

**Keywords:** Sodium nitrite, Enzyme inhibition, Natriuresis, Aquaresis, Central blood pressure

## Abstract

**Background:**

Sodium nitrite (NaNO_2_) causes vasodilation, presumably by enzymatic conversion to nitric oxide (NO). Several enzymes with nitrite reducing capabilities have been discovered in vitro, but their relative importance in vivo has not been investigated. We aimed to examine the effects of NaNO_2_ on blood pressure, fractional sodium excretion (FE_Na_), free water clearance (C_H2O_) and GFR, after pre-inhibition of xanthine oxidase, carbonic anhydrase, and angiotensin-converting enzyme. The latter as an approach to upregulate endothelial NO synthase activity.

**Methods:**

In a double-blinded, placebo-controlled, crossover study, 16 healthy subjects were treated, in a randomized order, with placebo, allopurinol 150 mg twice daily (TD), enalapril 5 mg TD, or acetazolamide 250 mg TD. After 4 days of treatment and standardized diet, the subjects were examined at our lab. During intravenous infusion of 240 μg NaNO_2_/kg/hour for 2 h, we measured changes in brachial and central blood pressure (BP), plasma cyclic guanosine monophosphate (P-cGMP), plasma and urine osmolality, GFR by ^51^Cr-EDTA clearance, FE_Na_ and urinary excretion rate of cGMP (U-cGMP) and nitrite and nitrate (U-NO_x_). Subjects were supine and orally water-loaded throughout the examination day.

**Results:**

Irrespective of pretreatment, we observed an increase in FE_Na_, heart rate, U-NO_x_, and a decrease in C_H2O_ and brachial systolic BP during NaNO_2_ infusion. P-cGMP and U-cGMP did not change during infusion. We observed a consistent trend towards a reduction in central systolic BP, which was only significant after allopurinol.

**Conclusion:**

This study showed a robust BP lowering, natriuretic and anti-aquaretic effect of intravenous NaNO_2_ regardless of preceding enzyme inhibition. None of the three enzyme inhibitors used convincingly modified the pharmacological effects of NaNO_2_. The steady cGMP indicates little or no conversion of nitrite to NO. Thus the effect of NaNO_2_ may not be mediated by NO generation.

**Trial registration:**

EU Clinical Trials Register, 2013-003404-39. Registered December 3 2013.

## Background

Sodium nitrite (NaNO_2_) has a well known vasodilatory effect, which is believed to rely on the enzymatic and non-enzymatic reduction of nitrite to nitric oxide (NO). The generation of NO from endogenous and exogenous nitrite is an alternative and parallel pathway to the classical synthesis of NO from L-arginine by endothelial NO synthase (eNOS). Several enzymes e.g. xanthine oxidase (XO), carbonic anhydrase (CA), and even eNOS, are reported to possess nitrite reducing capabilities, and hence increasing the bioavailability of NO using nitrite as substrate. Reduction of nitrite to NO occurs preferentially during hypoxia and acidosis, but most of the enzymes retain the ability to generate NO at physiological conditions, although at a lower rate and requiring higher concentrations of nitrite [1].

The XO inhibitor allopurinol has repeatedly been shown to attenuate the blood pressure (BP) reduction by NaNO_2_ in rats [2–4]. The effect of XO inhibition was selective to nitrite, as the vasodepressor effect of sodium nitroprusside, another NO donor, was intact in all three studies. Ghosh et al. found an association between nitrite reductase activity in erythrocytic XO and the efficacy of dietary nitrate to reduce BP in hypertensive patients [4].

Several studies have found favorable changes in NO metabolites during treatment with various inhibitors of angiotensin-converting enzyme (ACE). The mechanism is suggested to be an up-regulation of eNOS, due to an accumulation of bradykinin [5, 6]. It appears to be a class-effect of ACE inhibitors, as Comini et al. found a consistent increase in rat plasma NO_x_ (combined nitrate and nitrite), eNOS expression and eNOS activity using a range of ACE inhibitors, including enalapril [7]. In clinical trials, long-term treatments with lisinopril [8, 9] and perindopril [10] were found to elevate plasma levels of NO_x_ in hypertensive patients. Similarly, injection of quinaprilat in healthy subjects [11] and short-term treatment of normotensive type 1 diabetics with enalapril [12], were shown to enhance endothelial function evaluated by flow-mediated vasodilation.

In vitro studies have shown nitrite reducing effects of CA, and suggest a stimulatory effect of acetazolamide on NO generation, despite the inhibition of CO_2_ hydration [13, 14]. A recent clinical trial found a positive effect of nitrate intake on the increase in visually stimulated cerebral blood flow when injecting acetazolamide in healthy male subjects [15].

The relative significance of the individual nitrite reductases for the bioactivation of NaNO_2_ in vivo has yet to be determined. In this randomized, double-blind, 4-way crossover study we aimed to investigate the relative importance of ACE, XO, and CA for the various effects of NaNO_2_ under physiological conditions. After preceding enzyme modulation with allopurinol, enalapril, or acetazolamide, we measured the effects of NaNO_2_ infusion on 1) the central and brachial BP, 2) the renin-angiotensin-aldosterone system 3) plasma and urinary NO_x_ and guanosine 3′,5′-cyclic monophosphate (cGMP), and 4) the renal water and sodium regulation. We hypothesized the following: Pretreatment with allopurinol inhibits the ability of XO to reduce nitrite to bioactive NO and hence attenuates the effects of NaNO_2_, while enalapril and acetazolamide, on the contrary, stimulate the generation of NO from nitrite through eNOS and CA respectively, augmenting the effects of NaNO_2_.

## Methods

### Subjects

Subjects were recruited by advertisement on local educational institutions. Prior to enrollment, all subjects passed an examination including medical history, physical examination, office BP measurement, urine dipstick, electrocardiography, and the following blood samples: P-cholesterol, P-alkaline phosphatase, P-alanine aminotransferase, P-bilirubin, B-glycated hemoglobin (hemoglobin A1c), P-thyroid-stimulating hormone, P-urate, P-total CO_2_ in venous blood, P-sodium, P-potassium, P-creatinine, P-albumin, B-platelets, B-leukocytes, B-hemoglobin, and hematocrit.

### Inclusion criteria

Both gender, age 18–40 years, BMI 18.5–30.0 kg/m^2^.

### Exclusion criteria

Alcohol consumption > 14 drinks per week for women and > 21 drinks per week for men, smoking, substance abuse, current use of medicine except contraception, known intolerance to the study drugs, office BP > 140/90, diabetes mellitus, anemia, estimated GFR < 60 ml/min (MDRD), history or signs of clinically relevant kidney, heart, liver, lung, neurological, or endocrine diseases, pregnancy or lactation, and blood donation within 1 month of the first investigation.

### Withdrawal criteria

Development of exclusion criteria, serious or unacceptable adverse events, suspicion of poor compliance to study medication, sustained BP < 90/50 or symptoms of low BP during NaNO_2_ infusion.

### Design

The study was conducted as a double-blinded, placebo-controlled, 4-way crossover trial. Subjects received allopurinol, enalapril, acetazolamide, or placebo for 4 days in random order. Each treatment period was followed by an examination day. The examination days were separated by a wash-out period of at least 3 weeks.

### Study drugs

Allopurinol (Tablet Allopurinol “DAK”, 300 mg) was produced by Takeda Pharma A/S, Taastrup, Denmark. Enalapril (Tablet Enalapril “Actavis”, 5 mg) was produced by Actavis Nordic A/S, Gentofte, Denmark. Acetazolamide (Tablet Diamox, 250 mg) was produced by Goldshield Pharmaceuticals Limited, Surrey, United Kingdom. Placebo contained 120 mg of potato starch and 51 mg lactose monohydrate. All tablets were covered in opaque gelatine capsules and were identical in appearance. Sodium nitrite 10 mg/ml (Skanderborg Pharmacy, Denmark) was diluted in isotonic saline immediately before administration according to subject weight.

### Randomization

Treatment order was allocated consecutively at the time of inclusion by the principal investigator according to a randomization plan created on randomization.com by the Hospital Pharmacy, Central Denmark Region. Medication was packed, sealed and labeled by the Hospital Pharmacy. Investigators, lab technicians, and subjects were blinded to treatment order for the duration of the trial.

### Number of subjects

Using a power of 80% and a significance level of 5% the minimum number of subjects should be 14 when the minimum relevant relative change in fractional sodium excretion (FE_Na_) is 10%, and the standard deviation is 12%. Due to expected dropouts and incomplete voiding, the minimum number of included subjects was set to 20.

### Experimental procedure

#### Before examination

For 4 days prior to each examination day, subjects ingested a standard diet prepared by the hospital kitchen. One of two diet sizes, 11.000 kJ per day or 15.000 kJ per day, was chosen according to the estimated energy demands for each subject based on weight and physical activity. Regardless of diet size, the nutritional composition was 55% carbohydrates, 30% fat, and 15% protein, sodium content was 135 mmol per day and content of nitrate and nitrite was minimized. Subjects were asked to drink 2.5 L daily, including a maximum of two small cups of coffee or tea. No alcohol or soft drinks were allowed. The subjects were asked to take the study medication twice daily (TD) between 7–8 AM and 6–8 PM, with the last dose on the morning of the examination day. Dosage: Allopurinol: 150 mg TD, enalapril: 5 mg TD, acetazolamide: 250 mg TD.

#### Examination day

On each examination day, the subjects arrived at the lab at 7.45 AM after an overnight fast, bringing a 24-h urine collection. Two indwelling catheters were placed in antecubital veins, one for sequential blood samples and one in the opposite arm for the administration of chromium-51 labeled ethylenediamine tetraacetic acid (^51^Cr-EDTA) and NaNO_2_. An oral load of 175 ml of tap water every half hour was started at 7.30 AM with the last dose of study medication. The subjects were installed in supine position in a quiet, temperature-controlled (22–25 °C) room throughout the day. Voiding was done sitting or standing. After 90 min of adaptation, blood and urine samples were collected every 30 min from 9.30 AM to 1.30 PM. The first two clearance periods were used as baseline. The baseline periods were followed by four periods from 10.30 AM to 12.30 PM, during which a sustained infusion of 50 ml saline diluted NaNO_2_, 240 μg/kg/hour (= 3.48 μmol/kg/hour), was administered, and finally two post-infusion periods from 12.30 PM to 1.30 PM. Blood samples were analyzed for ^51^Cr-EDTA, P-sodium, and P-osmolality (P-Osm). The sample at 1.30 PM was analyzed for combined nitrite and nitrate (P-NO_x_) and the samples at 10.30 AM, 11.30 AM and 12.30 PM also for plasma concentrations of renin (PRC), arginine vasopressin (P-AVP), angiotensin II (P-ANGII), P-aldosterone, P-cGMP, P-urate and plasma total carbon dioxide in venous blood (P-(vB)-total CO_2_). The sample volume was replaced 1:1 with isotonic saline. Urine samples were analyzed for sodium, creatinine, osmolality (U-Osm), ^51^Cr-EDTA, γ-subunit of the epithelial sodium channel (U-ENaCγ), and aquaporin-2 (U-AQP2). Samples at 10.30 AM, 11.30 AM, 12.30 PM and 1.30 PM were furthermore analyzed for U-cGMP and U-NO_x_.

### Blood pressure measurements

Brachial blood pressure was measured oscillometrically using Omron 705IT (Omron Healthcare Co. Ltd., Kyoto, Japan) every 15 min. Central systolic blood pressure (cSBP) was estimated by tonometric pulse wave analysis HealthSTATS BPro (HealthSTATS International, Singapore). The device was applied and calibrated according to the directions of the manufacturer, using the averaged last three of four consecutive measurements with the Omron 705IT. Sequential measurements were made every 15 min from 9 AM to 1.30 PM.

### Renal function

Glomerular filtration rate (GFR) was measured by the constant infusion clearance technique with ^51^Cr-EDTA as reference substance [16].

### Biochemical analyses

Blood samples were drawn from an indwelling catheter, placed in ice water and centrifuged immediately at 2200 G for 10 min at 4 °C. Immediately after voiding, urine was centrifuged at 2200 G for 10 min at 4 °C. Concentrations of sodium, potassium, and creatinine were measured using routine methods at the Department of Clinical Biochemistry, Regional Hospital West Jutland, Denmark. Urine supernatant and plasma were kept frozen in cryotubes at − 80 °C (PRC, P-aldosterone, P-AVP, and combined nitrate and nitrite (P-NO_x_ and U-NO_x_)), or at − 20 °C (P-ANGII, P-cGMP, U-cGMP, U-ENaCγ, U-AQP2, P-Osm, and U-Osm) until assayed.

P-AVP and P-ANGII were extracted from plasma with C_18_ Sep-Pak (Waters Corporation, Milford, MA, USA) and determined by radioimmunoassay (RIA) as previously described [16, 17]. The antibodies against AVP were a gift from Professor Jacques Dürr (Miami, FL, USA). Minimal detection level: 0.5 pmol/L. Coefficients of variation: 13% (inter-assay) and 9% (intra-assay). Antibodies against ANGII were obtained from the Department of Clinical Physiology, Glostrup Hospital, Denmark. Minimal detection level: 2 pmol/L. Coefficients of variation: 12% (inter-assay) and 8% (intra-assay).

P-Aldosterone was determined by RIA (Demeditec Diagnostics GmbH, Kiel, Germany). The minimal detection level was 3.99 pmol/L. The coefficients of variations were 17.2% (inter-assay) and 12.6% (intra-assay).

Plasma renin concentration (PRC) was determined by a RIA kit from Cisbio Bioassays, Codolet, France. The minimal detection level: 1 pg/ml. Coefficients of variation in the range 4–263 pg/ml: 3.6–5.0% (interassay) and 0.9–3.6% (intra-assay).

Plasma and urine osmolality was determined by freeze-point depression (A_2_O Advanced Automated Osmometer, Advanced Instruments, MA, USA).

U-NO_x_ and P-NO_x_ were determined by a colorimetric assay (R&D Systems, Minneapolis, MN, USA). Nitrate was reduced to nitrite by nitrate reductase and subsequently converted to a deep purple azo compound by addition of Griess reagent. The concentration was determined by photometric measurement of absorbance at 540 nm. Minimal detection level in urine: 15.6 μmol/L and in plasma: 6.2 μmol/L. The coefficients of variation were 3.9% (inter-assay) and 1.8% (intra-assay).

U-cGMP and P-cGMP were determined by a competitive enzyme immunoassay kit from R&D Systems, Minneapolis, MN, USA. Minimal detection level: 1.14 pmol/L. Coefficients of variation: 6.9% (inter-assay) and 4.9% (intra-assay).

U-AQP2 was determined by RIA as previously described [18, 19]. Rabbit anti-AQP2 antibodies were a gift from Professor Soren Nielsen and Professor Robert Fenton, The Water and Salt Research Center, Aarhus University, Denmark. Minimal detection level: 32 pg/tube. Coefficients of variation: 11.7% (inter-assay) and 5.9% (intra-assay).

U-ENaCγ was measured by RIA as previously described [20]. ENaCγ was synthesized and purchased by Lofstrand, Gaithersburg, Maryland, USA. The ENaCγ antibodies were a gift from Professor Soren Nielsen and Professor Robert Fenton, The Water and Salt Research Center, Aarhus University. It was raised against a synthetic peptide in rabbits, and affinity purified as previously described [21].

### Calculations

FE_Na_ was calculated using the formula (sodium clearance (C_Na_) / ^51^Cr-EDTA clearance × 100%). C_Na_ was calculated as (U-Na / P-Na x urine output rate (UOR)). Free water clearance (C_H2O_) was calculated as (UOR – osmolar clearance (C_osm_)). C_osm_ was calculated as (U-osmolality / P-osmolality x UOR). Creatinine clearance was calculated for 24-h urine as (urine volume x U-creatinine) / (P-creatinine x urine collection period).

### Statistical analysis

Statistical tests were performed in SPSS Statistics ver. 20 (IBM Corp., Armonk, NY, USA). All data was graphically evaluated for normality using Q-Q plots. Where logarithmic transformation could correct skewed data, parametric tests were performed on the transformed data. Statistics were performed using one-way repeated measures (RM) ANOVA for comparing over time within each pretreatment. A two-way RM ANOVA with time and pretreatment as within factors were used to test for interaction between pretreatment and time. Comparisons between placebo and pretreatments at baseline or between baseline and individual time points within groups were performed using paired *t*-test. When skewed data could not be normalized by log transformation, Friedman test and Wilcoxon signed-rank test were performed instead. Normal distributed data are presented as means with 95% confidence intervals, and non-parametric data as medians with inter-quartile ranges in brackets. Statistical significance was defined as *p* < 0.05. Pairwise comparisons with baseline were Bonferroni corrected.

## Results

### Demographics

**Fig. 1:**
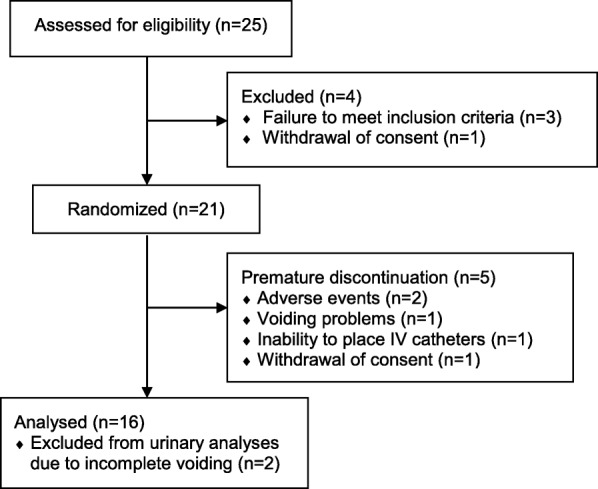
Subject flow in the study and reasons for exclusion

A total of 25 subjects were assessed for eligibility. Four were excluded due to elevated blood pressure (1), microscopic hematuria (1), elevated liver enzymes (1), and withdrawal of consent (1) prior to participation. During the study, five more dropped out, due to dizziness (1), headache (1), inability to void according to schedule (1), trouble placing intravenous catheters (1), and withdrawal of consent due to personal bustle (1) (Fig. 1). The remaining 16 completed the study; characteristics are presented in Table 1. Two was excluded from the urine analyses due to incomplete voiding during examination days.

**Table 1:** Clinical and laboratory characteristics of the 16 subjects

Gender (male/female)	5/11
Age (years)	23 [19;27]
BMI (kg/m^2^)	23 [20;29]
Systolic blood pressure (mmHg)	121 [111;132]
Diastolic blood pressure (mmHg)	73 [64;87]
P-alanine aminotransferase (U/l)	23 (16;28)
P-bilirubin (μmol/l)	8.0 (6.3;10.0)
P-alkaline phosphatase (U/l)	57 (44;82)
P-cholesterol (mmol/l)	4.2 [3.7;4.8]
B-glycated hemoglobin (mmol/mol)	34 (32;35)
P-thyroid stimulating hormone (mIE/l)	1.17 (0.78;2.05)
P-urate (mmol/l)	26 [22;30]
P-total CO_2_ in venous blood (mmol/l)	27 [26;28]
P-sodium (mmol/l)	140 [139;141]
P-potassium (mmol/l)	3.8 [3.7;3.9]
eGFR_MDRD_ (ml/min/1.73m^2^)	100 [92;107]
P-albumin (g/l)	42 [40;43]
B-platelets (×10^9^/l)	233 [208;259]
B-leukocytes (×10^9^/l)	6.3 [5.3;7.3]
B-hemoglobin (mmol/l)	8.6 [8.2;9.0]
Hematocrit	.40 [.42;.44]

Normal distributed data are presented as means with 95% confidence interval in brackets and non-parametric data as medians with 25th and 75th percentiles in parentheses. Estimated glomerular filtration rate (eGFR_MDRD_) is calculated using the Modification of Diet in Renal Disease Study equation

### Effect of pretreatment on baseline characteristics

As shown in Table 2, pretreatment with acetazolamide reduced creatinine clearance, absolute sodium excretion, and P-potassium, and increased fractional excretion of potassium (FE_K_) measured in 24-h urine. It decreased plasma total CO_2_ in venous blood and marginally increased P-urate. Pretreatment with allopurinol reduced P-urate, and slightly increased 24-h excretion of ENaCγ, while enalapril marginally increased 24-h excretion of albumin and slightly reduced P-sodium.

**Table 2:** Effect of allopurinol, enalapril and acetazolamide on 24-h urine collection and selected baseline blood samples

	Placebo	Allopurinol	Enalapril	Acetazolamide	P_RM ANOVA/Friedman_
CrCl (ml/min/1.73 m^2^)	134 [120;148]	137 [124;150]	130 [117;142]	116 [103;130]^*^	.030
Urine output (ml/min)	1.77 [1.42;2.12]	1.71 [1.38;2.03]	1.58 [1.20;1.96]	1.78 [1.48;2.08]	.411
C_H2O_ (ml/min)	−0.32 [−0.62;-0.01]	−0.43 [− 0.68;-0.17]	−0.36 [− 0.71;0.00]	−0.19 [− 0.49;0.10]	.304
P-sodium (mmol/l)	139 [138;139]	138 [137;139]	137 [137;138]^*^	138 [137;139]	.025
U-Na (mmol/24 h)	129 [107;151]	135 [115;154]	116 [98;134]	101 [80;122]^*^	.034
FE_Na_ (%)	0.47 [0.40;0.54]	0.49 [0.39;0.60]	0.45 [0.38;0.52]	0.42 [0.35;0.50]	.412
P-potassium (mmol/l)	4.1 [4.0;4.1]	4.0 [3.9;4.1]	4.1 [4.0;4.2]	3.6 [3.6;3.7]^*^	<.001
U-K (mmol/24 h)	57.8 (45.9;75.8)	59.2 (41.3;72.1)	51.9 (42.5;83.0)	76.3 (52.7;95.1)	.349
FE_K_ (%)	7.91 [6.71;9.11]	7.55 [6.17;8.92]	7.67 [6.48;8.85]	11.6 [9.84;13.4]^*^	<.001
U-albumin (mg/24 h)	2.98 (1.25;4.75)	2.02 (1.00;4.00)	4.50 (1.32;8.25)^*^	3.00 (2.25;4.75)	.184
U-AQP2 (ng/min)	0.51 (0.41;0.76)	0.64 (0.46;0.83)	0.62 (0.42;0.92)	0.50 (0.38;0.62)	.071
U-ENaCγ (ng/min)	0.23 (0.21;0.31)	0.28 (0.23;0.34)^*^	0.27 (0.23;0.36)	0.30 (0.24;0.38)	.091
P-urate (mmol/l)	0.29 [0.25;0.33]	0.16 [0.13;0.20]^*^	0.29 [0.25;0.33]	0.34 [0.30;0.38]^*^	<.001
P-(vB)-total CO_2_ (mmol/l)	25.1 [23.9;26.4]	25.0 [24.0;26.0]	24.4 [23.7;25.1]	18.4 [17.4;19.3]^*^	<.001

Effect of 4 days treatment with allopurinol, enalapril and acetazolamide on 24-h urine collection and selected baseline blood samples in 16 healthy subjects. Creatinine clearance (CrCl), urine output, free water clearance (C_H2O_), urinary excretion rate of sodium (U-Na) and potassium (U-K) and fractional excretion of sodium (FE_Na_) and potassium (FE_K_), urinary excretion rate of albumin, aquaporin-2 (U-AQP2), and γ-subunit of the epithelial sodium channel (U-ENaCγ), plasma sodium (P-Na), potassium (P-K), urate, and total carbon dioxide in venous blood (P-(vB)-total CO_2_). Normal distributed data are presented as means with 95% confidence interval in brackets, and non-parametric data as medians with 25th and 75th percentiles in parentheses. Statistics were performed using one-way repeated measures (RM) ANOVA or Friedman test (U-albumin, U-AQP2, U-ENaCγ). U-K was log transformed before RM ANOVA. Pairwise comparison with placebo was performed using Student’s *t*-test or Wilcoxon signed-rank test (U-albumin, U-AQP2, U-ENaCγ)

Statistically significantly different from placebo: ^*^ = *p* < .05

### Effect of NaNO_2_ on NO_x_ and cGMP

We measured a steady increase in P-NO_x_ and U-NO_x_ throughout the NaNO_2_ infusion, regardless of preceding enzyme inhibition. P-cGMP was unchanged during infusion after all pretreatments. U-cGMP did not change during the infusion (*p* > 0.05 vs. baseline) but decreased significantly in the post-infusion period (Table 3).

**Table 3:** Effect of intravenous NaNO_2_ on combined nitrate and nitrite (NOx) and cGMP

	Baseline	Infusion		Post-infusion	*p*_RM ANOVA (one-way)_
		60 min	120 min	180 min	
P-NO_x_ (μmol/l)					
Placebo	17 (13;21)	25 (19;31)^*^	31 (24;38)^*^	29 (22;35)^*^	<.001
Allopurinol	17 (14;23)	24 (19;30)^*^	32 (26;36)^*^	30 (26;32)^*^	<.001
Enalapril	15 (12;19)	24 (21;31)^*^	29 (26;32)^*^	27 (24;31)^*^	<.001
Acetazolamide	17 (13;19)	24 (21;31)^*^	30 (27;35)^*^	27 (23;32)^*^	<.001
*p*_interaction (pretreatment x time)_		.742			
U-NO_x_ (μmol/min)					
Placebo	0.52 (0.37;0.69)	0.72 (0.52;0.79)^*^	0.94 (0.88;1.09)^*^	0.95 (0.83;1.21)^*^	<.001
Allopurinol	0.60 (0.42;0.88)	0.79 (0.65;0.98)^*^	0.99 (0.87;1.23)^*^	1.07 (0.90;1.18)^*^	<.001
Enalapril	0.57 (0.44;0.78)	0.69 (0.59;0.97)^*^	0.99 (0.87;1.22)^*^	0.94 (0.80;1.31)^*^	<.001
Acetazolamide	0.49 (0.45;0.54)	0.63 (0.59;0.67)^*^	0.98 (0.86;1.05)^*^	0.94 (0.85;1.06)^*^	<.001
*p*_interaction (pretreatment x time)_		.677			
P-cGMP (pmol/ml)					
Placebo	88 [78;98]	93 [83;104]	87 [78;95]		.310
Allopurinol	91 [83;100]	87 [77;98]	88 [77;98]		.597
Enalapril	82 [71;92]	85 [74;96]	93 [75;110]		.102
Acetazolamide	90 [78;101]	84 [74;93]	84 [74;94]		.088
*p*_interaction (pretreatment x time)_		.111			
U-cGMP (pmol/min)					
Placebo	425 (356;530)	426 (371;561)	414 (341;515)	321 (235;343)^*^	<.001
Allopurinol	487 (388;613)	453 (353;575)	461 (376;570)	367 (287;443)^*^	<.001
Enalapril	404 (291;527)^†^	425 (329;552)	409 (309;486)	284 (243;349)^*^	<.001
Acetazolamide	361 (305;484)^†^	345 (296;468)	372 (325;470)	269 (236;324)^*^	<.001
*p*_interaction (pretreatment x time)_		.362			

Effect of intravenous NaNO_2_ on plasma concentrations (*n* = 16) and urinary excretion rates (*n* = 14) of combined nitrate and nitrite (NO_x_) and guanosine 3′,5′-cyclic monophosphate (cGMP) in healthy subjects after 4 days pretreatment with allopurinol, enalapril, acetazolamide, or placebo. Normal distributed data are presented as means with 95% confidence interval in brackets and non-parametric data as medians with 25th and 75th percentiles in parentheses. Statistics were performed using one-way repeated measures (RM) ANOVA for comparing over time and two-way RM ANOVA with time and pretreatment as within factors to test for interaction. Pairwise comparison where performed using Student’s *t*-test. P-NO_x_, U-NO_x_ and U-cGMP were log transformed prior to testing

^*^ *p* < .05 within group vs. baseline (Bonferroni), ^†^ *p* < .05 vs. placebo at baseline

### Effect of NaNO_2_ on GFR and renal sodium and water excretion

As shown in Table 4, baseline GFR was significantly lower after acetazolamide compared to placebo (*p* < 0.001), while enalapril and allopurinol did not change baseline GFR. Infusion of NaNO_2_ did not alter GFR besides transient fluctuations after placebo and acetazolamide. The baseline reduction of GFR after acetazolamide was sustained throughout the examination day. None of the pretreatments changed baseline fractional sodium excretion (FE_Na_) compared to placebo. During NaNO_2_ infusion, FE_Na_ increased regardless of pretreatment. Urinary sodium excretion increased in a similar way as FE_Na_ (data not presented). Excretion rate of ENaCγ was significantly higher at baseline and during NaNO_2_ infusion after acetazolamide, while the median level of excretion rate did not change consistently during infusion after any of the pretreatments.

**Table 4:** Effect of intravenous NaNO_2_ on renal sodium and water regulation

	Baseline	Infusion				Post-infusion		*p*_Friedman/RM ANOVA (one-way)_
		30 min	60 min	90 min	120 min	150 min	180 min	
FE_Na_ (%)								
Placebo	1.26 [1.05;1.47]	1.26 [1.09;1.43]	1.43 [1.25;1.62]	1.38 [1.24;1.52]	1.57 [1.36;1.78]	1.53 [1.34;1.73]	1.41 [1.26;1.56]	.001
Allopurinol	1.36 [0.96;1.75]	1.41 [1.05;1.76]	1.52 [1.20;1.83]	1.53 [1.22;1.83]	1.66 [1.32;2.00]^*^	1.69 [1.40;1.98]^*^	1.59 [1.33;1.85]	.002
Enalapril	1.15 [0.84;1.46]	1.30 [1.01;1.60]	1.42 [1.15;1.68]^*^	1.45 [1.20;1.71]^*^	1.59 [1.34;1.83]^*^	1.56 [1.32;1.81]^*^	1.52 [1.29;1.74]^*^	<.001
Acetazolamide	1.08 [0.86;1.29]	1.25 [1.01;1.49]	1.35 [1.08;1.63]	1.29 [1.07;1.51]	1.42 [1.15;1.69]^*^	1.44 [1.17;1.71]^*^	1.28 [1.05;1.52]	.001
*p*_interaction (pretreatment x time)_		.535						
GFR (ml/min/1.73 m^2^)								
Placebo	98 [90;105]	104 [96;111]	98 [91;105]	105 [97;113]^*^	101 [92;110]	99 [93;106]	100 [91;109]	.027
Allopurinol	99 [90;108]	102 [93;112]	97 [89;105]	103 [94;112]	98 [88;108]	99 [91;107]	102 [92;111]	.132
Enalapril	101 [92;110]	104 [94;114]	101 [94;108]	104 [96;112]	100 [91;109]	102 [94;109]	102 [94;110]	.568
Acetazolamide	83 [76;89]^†^	84 [77;92]	77 [70;83]^*^	83 [75;91]	83 [76;89]	83 [76;89]	85 [78;92]	.012
*p*_interaction (pretreatment x time)_		.494						
C_H20_ (ml/min)								
Placebo	4.58 [3.66;5.50]	2.87 [2.08;3.65]	1.87 [1.36;2.38]^*^	2.27 [1.46;3.08]^*^	2.85 [2.11;3.59]	2.64 [2.00;3.28]^*^	2.85 [2.15;3.55]^*^	<.001
Allopurinol	4.56 [3.64;5.48]	3.05 [2.02;4.07]	2.54 [1.82;3.27]^*^	2.04 [0.98;3.10]^*^	3.00 [2.11;3.90]	3.06 [2.19;3.93]	3.26 [2.40;4.12]	.011
Enalapril	4.82 [3.70;5.94]	3.57 [2.48;4.65]	2.28 [1.26;3.29]^*^	2.28 [1.48;3.08]^*^	3.24 [2.59;3.89]	2.23 [1.26;3.21]^*^	2.67 [1.71;3.63]	.001
Acetazolamide	3.91 [2.80;5.01]	1.99 [1.12;2.87]	1.69 [0.93;2.46]	1.63 [0.87;2.39]^*^	2.60 [1.78;3.42]	2.67 [1.78;3.55]	2.60 [1.80;3.40]	.004
*p*_interaction (pretreatment x time)_		.682						
P-AVP (pg/ml)								
Placebo	0.32 [0.25;0.38]		0.25 [0.20;0.30]^*^		0.27 [0.21;0.33]^*^			.012
Allopurinol	0.29 [0.21;0.36]		0.28 [0.22;0.33]		0.25 [0.21;0.29]			.414
Enalapril	0.26 [0.20;0.33]		0.21 [0.17;0.26]		0.23 [0.18;0.28]			.213
Acetazolamide	0.29 [0.22;0.36]		0.27 [0.19;0.35]		0.28 [0.22;0.34]			.770
*p*_interaction (pretreatment x time)_		.630						
U-AQP2 (ng/min)								
Placebo	1.26 [1.12;1.41]	1.25 [1.06;1.45]	1.23 [1.10;1.35]	1.18 [1.04;1.33]	1.23 [1.08;1.39]	1.19 [1.06;1.33]	1.13 [1.02;1.23]	.167
Allopurinol	1.45 [1.27;1.63]^†^	1.37 [1.26;1.48]	1.31 [1.20;1.42]	1.34 [1.21;1.47]	1.33 [1.13;1.54]	1.31 [1.20;1.42]	1.28 [1.17;1.39]	.080
Enalapril	1.29 [1.13;1.44]	1.23 [1.09;1.37]	1.28 [1.14;1.42]	1.26 [1.13;1.40]	1.28 [1.09;1.46]	1.33 [1.18;1.48]	1.18 [1.06;1.31]	.131
Acetazolamide	1.30 [1.15;1.45]	1.26 [1.08;1.44]	1.21 [1.07;1.36]	1.19 [1.04;1.35]	1.25 [1.09;1.41]	1.26 [1.08;1.43]	1.21 [1.06;1.37]	.363
*p*_interaction (pretreatment x time)_		.590						
U-ENaCγ (ng/min)								
Placebo	0.44 (0.38;0.51)	0.44 (0.33;0.51)	0.47 (0.35;0.54)	0.45 (0.36;0.59)	0.40 (0.36;0.57)	0.43 (0.36;0.56)	0.43 (0.32;0.46)	.331
Allopurinol	0.43 (0.40;0.65)	0.42 (0.38;0.54)	0.39 (0.34;0.54)	0.47 (0.42;0.61)	0.43 (0.30;0.54)^*^	0.42 (0.35;0.51)	0.39 (0.34;0.50)	.020
Enalapril	0.44 (0.40;0.64)	0.40 (0.35;0.49)	0.39 (0.34;0.60)	0.45 (0.31;0.53)	0.39 (0.36;0.55)	0.41 (0.33;0.57)	0.45 (0.40;0.53)	.215
Acetazolamide	0.68 (0.48;0.95)^†^	0.57 (0.42;1.01)	0.59 (0.45;0.94)	0.53 (0.48;0.88)	0.55 (0.45;0.93)	0.71 (0.53;0.94)	0.64 (0.49;0.89)	.561

Effect of intravenous NaNO_2_ on fractional excretion of sodium (FE_Na_), GFR, free water clearance (C_H2O_), urinary excretion rates of aquaporin-2 (AQP2) and γ-subunit of the epithelial sodium channel (ENaCγ) in 14 healthy subjects and arginine vasopressine (AVP) in 16 healthy subjects after 4 days pretreatment with allopurinol, enalapril, acetazolamide, or placebo. Normal distributed data are presented as means with 95% confidence interval in brackets and non-parametric data as medians with 25th and 75th percentiles in parentheses. Statistics were performed using one-way repeated measures (RM) ANOVA for comparing over time and two-way RM ANOVA with time and pretreatment as within factors to test for interaction. Pairwise comparison where performed using Student’s *t*-test. U-ENaCγ were tested using Friedman test for comparing over time and Wilcoxon’s signed rank test for pairwise comparison with placebo or baseline

^*^ *p* < .05 within group vs. baseline (Bonferroni), ^†^ *p* < .05 vs. placebo at baseline

At baseline, there was no significant difference in C_H2O_, or urine output (UO, not presented) between the pretreatments. During and after NaNO_2_ infusion, we observed a decrease in C_H2O_ and UO (not presented), with the maximum effect after 60–90 min. This response was not modified by any of the pretreatments. At baseline, U-AQP2 was significantly higher after allopurinol, while there was no difference in P-AVP regardless of pretreatment. In response to NaNO_2_, we observed a significant decrease in P-AVP on the placebo day. The reduction was insignificant after allopurinol, enalapril, and acetazolamide. We could not detect significant changes in U-AQP2 in response to NaNO_2_, neither after placebo nor after preceding enzyme inhibition.

### Effect of NaNO_2_ on blood pressure

As shown in Table 5, brachial systolic BP was decreased, and heart rate increased during NaNO_2_ infusion regardless of pretreatment. The reduction in brachial diastolic BP was only significant after acetazolamide (*p* = 0.035). Brachial mean arterial pressure (MAP) was significantly reduced in response to NaNO_2_ infusion regardless of pretreatment. There was a trend to a reduction in central systolic BP, which was only significant after allopurinol (*p* = 0.047).

**Table 5:** Effect of intravenous NaNO_2_ on brachial and central hemodynamics

	Baseline value	Change from baseline to last hour of infusion	*p*_*t*-test_
Brachial systolic BP (mmHg)			
Placebo	115 (111;119)	−2.63 (− 4.41;-0.85)	.007
Allopurinol	115 (111;119)	− 3.07 (−5.12;-1.01)	.006
Enalapril	110 (107;114)^†^	−3.84 (−5.32;-2.35)	<.001
Acetazolamide	114 (111;118)	−3.79 (−5.85;-1.74)	.001
*p*_RM ANOVA_		.272	
Brachial diastolic BP (mmHg)			
Placebo	60 (57;62)	−1.50 (−3.28;0.28)	.093
Allopurinol	60 (56;63)	−1.22 (−3.14;0.70)	.196
Enalapril	56 (53;59)^†^	−1.18 (−2.40;0.03)	.054
Acetazolamide	60 (58;63)	−1.58 (−3.04;-0.12)	.035
*p*_RM ANOVA_		.923	
Brachial MAP (mmHg)			
Placebo	78 (76;81)	−1.88 (−3.39;-0.37)	.018
Allopurinol	78 (75;81)	−1.84 (−3.63;-0.04)	.046
Enalapril	74 (72;77)^†^	−2.07 (−3.28;-0.86.)	.002
Acetazolamide	78 (76;81)	−2.32 (−3.88;-0.76)	.006
*p*_RM ANOVA_		.848	
Heart rate (beats per minute)			
Placebo	55 (52;58)	3.37 (1.95;4.79)	<.001
Allopurinol	55 (52;59)	1.70 (0.12;3.28)^†^	.037
Enalapril	56 (52;60)	2.21 (0.97;3.45)	.002
Acetazolamide	57 (53;61)	1.30 (0.11;2.50)^†^	.035
*p*_RM ANOVA_		.050	
Central systolic BP (mmHg)			
Placebo	98 (89;106)	−1.38 (−6.03;3.26)	.528
Allopurinol	99 (91;107)	−4.51 (−8.95;-0.07)	.047
Enalapril	97 (88;106)	−4.04 (−8.13;0.05)	.053
Acetazolamide	101 (90;112)	−2.69 (− 8.30;2.91)	.320
*p*_RM ANOVA_		.804	

Effect of intravenous NaNO_2_ on heart rate, brachial and central blood pressure (BP) in 16 healthy subjects after 4 days pretreatment with allopurinol, enalapril, acetazolamide, or placebo. Data are means with 95% confidence interval in brackets. Baseline values are an average of measurements in the one-hour period prior to infusion. The baseline values were compared to an average of measurements during the last hour of NaNO_2_ infusion. Pairwise comparisons were performed using Student’s *t*-test. One-way repeated measures (RM) ANOVA was used for comparison of effects between pretreatments

^†^: *p* < .05 vs. placebo

### Effect of NaNO_2_ on the renin-angiotensin-aldosterone system (RAAS)

As depicted in Table 6, PRC was clearly increased at baseline after pretreatment with enalapril, and to a lesser extent after acetazolamide, compared to placebo. In response to NaNO_2_ infusion, PRC increased further after both enalapril and acetazolamide. Compared to placebo, angiotensin II (AngII) was decreased at baseline after enalapril and allopurinol. In response to NaNO_2_ infusion, AngII increased marginally only after acetazolamide. Aldosterone was increased at baseline after acetazolamide compared to placebo. The baseline suppression after enalapril was insignificant compared to placebo (*p* = 0.079). NaNO_2_ infusion did not affect aldosterone levels after any of the pretreatments.

**Table 6:** Effect of intravenous NaNO_2_ on the renin-angiotensin-aldosteron system

	Baseline	Infusion		*p*_RM ANOVA (one-way)_
		60 min	120 min	
PRC (pg/ml)				
Placebo	9 (6;14)	9 (7;16)	9 (8;16)	.262
Allopurinol	9 (5;12)	9 (6;16)^*^	10 (6;14)	.024
Enalapril	54 (27;89)^†^	72 (40;93)^*^	69 (61;95)^*^	.014
Acetazolamide	15 (9;20)^†^	16 (12;23)	18 (12;23)^*^	.008
*p*_interaction (pretreatment x time)_		.168		
P-AngII (pg/ml)				
Placebo	16 (12;22)	17 (13;20)	14 (11;23)	.239
Allopurinol	14 (8;19)^†^	14 (12;21)	13 (10;19)	.175
Enalapril	11 (6;17)^†^	13 (8;21)	11 (8;21)	.189
Acetazolamide	19 (11;34)	20 (14;34)^*^	21 (16;35)^*^	.001
*p*_interaction (pretreatment x time)_		.160		
P-Aldo (pmol/l)				
Placebo	128 (98;161)	121 (90;155)	106 (79;155)	.228
Allopurinol	105 (82;158)	98 (64;138)	113 (76;147)	.967
Enalapril	78 (56;106)	77 (54;97)	71 (59;117)	.511
Acetazolamide	184 (132;281)^†^	178 (125;308)	230 (110;352)	.826
*p*_interaction (pretreatment x time)_		.664		

Effect of intravenous NaNO_2_ on plasma concentrations of renin (PRC), angiotensin II (P-AngII), and aldosterone (P-Aldo) in 16 healthy subjects after 4 days pretreatment with allopurinol, enalapril, acetazolamide, or placebo. Data are medians with 25th and 75th percentiles in parentheses. After log transformation, statistics were performed using one-way repeated measures (RM) ANOVA for comparing over time and two-way RM ANOVA with time and pretreatment as within factors to test for interaction. Pairwise comparison with placebo or baseline where performed using Student’s *t*-test after log transformation

^*^ *p* < .05 within group vs. baseline (Bonferroni), ^†^ *p* < .05 vs. placebo at baseline

### Safety

We observed 26 adverse events, predominantly expected side effects such as paresthesia after acetazolamide (7), gastrointestinal discomfort after enalapril (5), headache (8), and lightheadedness (3) including one event of micturition syncope. None of the adverse events were considered serious.

## Discussion

In the present study, our aim was to investigate the impact of preceding short-term modulation of three different enzyme systems on the various acute effects of sodium nitrite infusion. Without active pretreatment, we found that intravenous NaNO_2_ led to an increased natriuresis, an increase in P-NO_x_ and U-NO_x_, a small decrease in AVP, and a systolic BP reduction, along with a decreased aquaresis, an unaffected P-cGMP and even a reduction in U-cGMP in the post-infusion period. Preceding enzyme inhibition did not convincingly modify the effects.

The decrease we observed in brachial BP is in agreement with other studies using comparable doses [22–24]. However, this reduction is smaller than in a recent dose-response study, where our group in addition to a larger decrease in brachial systolic and mean arterial BP found a significant reduction in both the brachial diastolic and the central systolic BP using the same dose of NaNO_2_ [25]. The reason for this discrepancy might be the different gender ratio in the two studies; the percentage of females was 69% in the present study compared to 42% in the dose-response study. While no gender difference has been reported for NaNO_2_, Kapil et al. found a substantially greater reduction in both systolic and diastolic brachial BP in males compared to females when ingesting potassium nitrate [26].

We observed an increase in both fractional and absolute sodium excretion during NaNO_2_ infusion. Although there is not complete agreement on the effects of NO in different nephron segments, a net natriuretic and diuretic effect of NO in vivo is commonly accepted [27]. The natriuretic effect we observed during NaNO_2_ infusion is in agreement with existing data regarding the overall inhibitory effect of NO on sodium absorption in the nephron. However, we found a reduction in free water clearance and urine output (data not presented), which conflicts with the general notion of NO as a diuretic. A possible explanation could be the reduction in BP. The decrease in P-AVP and steady U-AQP2 suggests that the mechanism is not mediated by AQP2.

Soluble guanylyl cyclase (sGC) releases cGMP to the circulation upon stimulation by NO. Being a renowned second messenger for NO, cGMP is widely used as a surrogate marker of NO activity [28–30]. The lack of increase in P-cGMP and the post-infusion decline in U-cGMP in the present study is puzzling. The findings are nevertheless in agreement with a recent dose-response study from our group [25]. Accordingly, Omar et al. found that an accumulated intra-arterial infusion of approximately 100 mg of NaNO_2_ did not increase systemic cGMP, measured in the contralateral arm, despite a substantial increase in regional cGMP formation and an 11 mmHg reduction in MAP [31]. In comparison, the accumulated dose used in the present study was approximately 32 mg for the average subject weighing 67 kg.

Allopurinol reduced P-urate at baseline, as expected. While NaNO_2_ infusion lowered the brachial BP without active pretreatment, the reduction in central systolic BP (cSBP) was only significant after pretreatment with allopurinol. The cSBP reduction was not significantly different to the reduction after placebo pretreatment (*p* = 0.539), partly owing to an inherent lesser precision in the tonometry based method [32]. Although the results should be interpreted cautiously, this could indicate a potentiation of the vasodilating effects of NaNO_2_. The remaining effects of NaNO_2_ were unaffected by pretreatment with allopurinol. The intact, or even potentiated, vasodilating effect of NaNO_2_ after allopurinol is in agreement with the findings by Dejam et al. of an augmented increase in forearm blood flow when NaNO_2_ and oxypurinol was co-infused [23]. A possible explanation could be a reduced scavenging of NO due to inhibition of xanthine oxidase-generated reactive oxygen species.

We observed an increase in renin concentration during NaNO_2_ infusion, but only after preceding stimulation of renin secretion by either enalapril or to a lesser extent acetazolamide. The interplay between NO and renin has been studied intensively since the late 1980’s. Numerous in vitro studies have shown both inhibitory [33, 34], stimulatory [35, 36] and even biphasic effects [37] of NO or cGMP on renin secretion, while most in vivo studies agree on a stimulatory effect [38–40]. Our findings are in agreement with previous studies from our lab, showing a reduction of renin secretion after systemic NO inhibition in healthy subjects with activated renin system at baseline due to sodium restriction [41] or after angiotensin II receptor blockade [42]. A confounding effect of the BP reduction, being the strongest mediator of renin release, cannot be completely ruled out. However, NaNO_2_ infusion only increased renin when the basal renin concentration was elevated, after enalapril or acetazolamide, despite a comparable effect on the BP regardless of pretreatment. This pattern corresponds to a previous study by our group, showing no stimulatory effect on the RAAS when infusing BP reducing doses of NaNO_2_ after moderate sodium intake [25]. Interestingly, the BP lowering effect of NaNO_2_ was fully preserved after preceding BP reduction with enalapril, suggesting that the BP reducing mechanism of NaNO_2_ is independent of ACE activity.

Acetazolamide had a profound effect on multiple baseline values, e.g. 24-h sodium excretion, 24-h creatinine clearance, baseline GFR, and excretion rate of ENaCγ. Previous studies have shown an acute diuretic and natriuretic effect [43, 44] in the proximal tubule, which waned off after a few days of continued treatment [44]. We detected an activation of the RAAS, with significantly elevated levels of renin and aldosterone, most likely caused by a decrease in extracellular volume and pH. Aldosterone, being the primary regulator of ENaC, was most probably responsible for the increase in ENaC and accompanying increase in sodium reabsorption. The decrease in GFR is well known and believed to be mediated by tubuloglomerular feedback [45]. The effects of NaNO_2_ infusion after acetazolamide did not differ from after placebo pretreatment.

We hypothesized 1) an attenuation of the effects of NaNO_2_ after inhibition of the nitrite reducing capabilities of XO with allopurinol as shown in rats [2–4], 2) an augmentation of the effects of NaNO_2_ after enalapril due to accumulation of bradykinin leading to up-regulation of eNOS [5, 6], and 3) an enhanced effect of NaNO_2_ after acetazolamide due to a stimulated enzymatic conversion of nitrite to NO by carbonic anhydrase [13, 14]. However, apart from a reduction of central systolic BP, which was only significant after allopurinol, we could not detect any consistent differences in the response to NaNO_2_ between the pretreatments. The results suggest that none of the studied pathways are essential to nitrite bioactivation.

The post-infusion reduction in U-cGMP and steady P-cGMP is consistent with our previous findings [25], but nevertheless puzzling and could indicate that the effects of NaNO_2_ might not be mediated by the NO-sGC-cGMP pathway. If the actions of NaNO_2_ under physiologic conditions are independent of NO production, it would explain why modulation of different enzyme systems with suspected nitrite reducing abilities failed to modify the effect. The differences in baseline parameters after each pretreatment were expected and can be ascribed to the fundamental effects of the enzyme inhibitors.

### Strengths and limitations

The strengths of the present study lie in the design. It is a rigorously conducted, double-blinded, placebo-controlled, 4-way crossover study. The sodium intake is standardized and controlled. Adherence to the pretreatment was verified by baseline levels of P-renin, P-urate, and P-(vB)-total CO_2_ which reflected the pretreatment for all subjects without exception. Previous studies from our laboratory [25, 29, 46, 47] suggest a slightly natriuretic and anti-aquaretic effect of the supine and water loaded model, which might have contributed to the findings in the present study.

The dosage of NaNO_2_ relies on a previous dose-response study by our group [25]. Evaluated on the effects on P-NO_x_, U-NO_x_, sodium excretion and BP, we believe to have achieved a relevant increase in nitrite bioavailability.

## Conclusion

This study demonstrated a robust BP lowering, natriuretic and anti-aquaretic effect of intravenous NaNO_2_ regardless of preceding enzyme inhibition. The steady P-cGMP and post-infusion decrease in U-cGMP indicates little or no conversion of nitrite to NO. Thus the effect of NaNO_2_ may not be mediated by NO generation.

## Abbreviations

^51^Cr-EDTA: Chromium-51 labeled ethylenediamine tetraacetic acid
ACE: Angiotensin-converting enzyme
Aldo: Aldosterone
ANGII: Angiotensin II
AQP2: Aquaporin-2
AVP: Arginine vasopressin
BP: Blood pressure
CA: Carbonic anhydrase
cGMP: Guanosine 3′,5′-cyclic monophosphate
C_H2O_: Free water clearance
C_Na_: Sodium clearance
C_osm_: Osmolar clearance
cSBP: Central systolic blood pressure
ENaCγ: γ-subunit of the epithelial sodium channel
eNOS: Endothelial nitric oxide synthase
FE_K_: Fractional excretion of potassium
FE_Na_: Fractional excretion of sodium
GFR: Glomerular filtration rate
MAP: Mean arterial pressure
NaNO_2_: Sodium nitrite
NO: Nitric oxide
NO_x_: Combined nitrite and nitrate
P-(vB)-total CO_2_: Plasma total carbon dioxide in venous blood
PRC: Plasma renin concentration
RIA: Radioimmunoassay
sGC: Soluble guanylyl cyclase
UO: Urine output
UOR: Urine output rate
XO: Xanthine oxidase
